# SAM Composition and Electrode Roughness Affect Performance of a DNA Biosensor for Antibiotic Resistance

**DOI:** 10.3390/bios9010022

**Published:** 2019-02-07

**Authors:** Adrian Butterworth, Elizabeth Blues, Paul Williamson, Milovan Cardona, Louise Gray, Damion K Corrigan

**Affiliations:** 1 Department of Biomedical Engineering, Wolfson Centre, 106 Rottenrow East, University of Strathclyde, Glasgow G1 1XQ, UK; adrian.butterworth@strath.ac.uk (A.B.); elizabeth.blues.2014@uni.strath.ac.uk (E.B.); paul.williamson@strath.ac.uk (P.W.); milovan.cardona@strath.ac.uk (M.C.); 2 FlexMedical Solutions, Eliburn Industrial Park, Livingston, EH54 6GQ, Scotland; louise.e.gray@gmail.com

**Keywords:** electrochemical biosensor, DNA detection, point-of-care diagnostics, electrochemical impedance spectroscopy, self-assembled monolayers (SAMs)

## Abstract

Antibiotic resistance is a growing concern in the treatment of infectious disease worldwide. Point-of-care (PoC) assays which rapidly identify antibiotic resistance in a sample will allow for immediate targeted therapy which improves patient outcomes and helps maintain the effectiveness of current antibiotic stockpiles. Electrochemical assays offer many benefits, but translation from a benchtop measurement system to low-cost portable electrodes can be challenging. Using electrochemical and physical techniques, this study examines how different electrode surfaces and bio-recognition elements, i.e. the self-assembled monolayer (SAM), affect the performance of a biosensor measuring the hybridisation of a probe for antibiotic resistance to a target gene sequence in solution. We evaluate several commercially available electrodes which could be suitable for PoC testing with different SAM layers and show that electrode selection also plays an important role in overall biosensor performance.

## 1. Introduction

The molecular detection of biomolecules through the application of electrochemical techniques has been widely studied due to the high sensitivity, specificity and simplicity of these methods [1]. DNA and antibody-based sensors are popular choices for analyte detection, taking advantage of their specific interactions to accurately identify the presence of a target in a sample. Both DNA and protein detection can be performed through the inclusion of redox reporters, such as DNA intercalators or electroactive enzyme substrates [2,3], through the labelling of the target or capture molecules [4,5], or through direct detection of the target’s effect on the interfacial properties of the electrode when under electrochemical measurement [6,7]. The latter methods do not require any modification of the target or probe and therefore offer a simple method for molecular detection. Assays based on label-free electrochemical methods typically involve tests which are highly sensitive to surface characteristics, and as such require well-characterised and homogenous surfaces in order to obtain reliable results. 

Modern diagnostics are tending towards point-of-care (PoC) tests: assays which can be performed at the bedside, in the field or at home. Such tests are often expected to provide results in minutes, unlike the centralised laboratory tests which may take hours to complete or require the collection of large sample numbers to be viable. PoC tests typically use disposable test cartridges or strips which limit opportunities for cross-contamination and minimise the amount of training and interaction required from the user. The electrodes typically used in benchtop electrochemical measurements are large, costly and require external reference and counter electrodes to complete the cell, making them unfeasible for use in a disposable PoC system. These electrodes also require extensive cleaning between uses and can exhibit memory effects after repeated tests. As a result, smaller, self-contained electrode platforms have been widely explored for assay development [8,9,10,11,12,13]. Electrodes which are low-cost and easily manufactured in large quantities are ideal for such PoC measurements.

These advantages make PoC sensors a promising technology to monitor and limit antibiotic resistance. The development and spread of antibiotic resistance is a growing concern, which could render our current stock of antibiotics ineffective against infection [14]. In particular, Gram-negative bacteria exhibit widespread resistance to a range of antibiotics; the latest surveillance from within the EU indicates that only 42% of reported *E. coli* isolates were fully susceptible to antibiotics, and some species such as *A. baumannii* have 43% of isolated resistance to 3 antibiotic classes, including carbapenems [15]. Some methods such as the implementation of antibiotic stewardship programs, increasing public understanding of antibiotics and reducing the use of antibiotics in livestock have been employed to reduce and control resistance [16]. Antibiotic stewardship in hospitals has been shown to reduce the prevalence of some resistant organisms, but the prevalence of resistant community-associated infections (especially in long term care facilities) mean that resistant organisms can be reintroduced into a clinical setting easily [17]. Biosensors employed at the bedside will allow clinicians to more rapidly identify antibiotic resistance and to prescribe tailored treatment to patients earlier in their care, which should in turn reduce opportunities for antibiotic resistance to spread and improve outcomes for the patient. 

**Figure 1:**
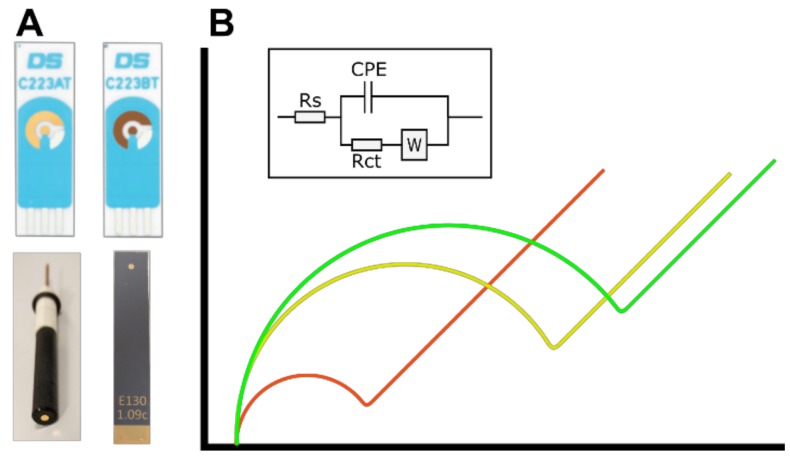
(**A**) Images of electrodes used in these experiments. From top left to bottom right; AT screen-printed electrode, BT screen-printed electrode, polycrystalline gold electrode, thin-film gold electrode. (**B**) Example impedance responses of electrodes which are clean (red), modified with a self-assembled monolayer (SAM) and probe layer (yellow), and with DNA target bound to the probe (green). Inset: the Randles equivalent circuit used to fit impedance data; Rs—solution resistance, CPE—constant phase element, Rct—charge transfer resistance, W—Warburg element.

Here, we examine the suitability of several electrode systems which show promise as disposable devices for PoC electrochemical testing (Figure 1A). Screen-printed electrodes (SPEs) were examined as they have been employed in a number of biological systems such as the detection of tumour biomarkers [18,19], thrombin levels [20], DNA damage [21], or pathogenic bacteria [22,23]. These electrodes also represent a low-cost (~£2.40) and scalable sensor for electrochemical detection. In this study, these are examined against traditional polycrystalline gold electrodes (PGEs) which are commonly used in benchtop electrochemistry, and thin-film gold electrodes (TFGEs) defined by a dielectric which represents a pure gold surface which can be mass produced for less than £2 per device. Cyclic voltammetry (CV) and electrochemical impedance spectroscopy (EIS) measurements are performed in order to characterise the electrodes when clean, functionalised with DNA probe and after incubation with a target sequence. CV is a standard electrochemical method which can be used as a cleaning process or as an analytical technique measuring redox reactions at an electrode surface [24]. EIS is a highly sensitive technique which can probe the interfacial characteristics of an electrode by measuring the impedance of the system at different frequencies [25]. A small alternating current is applied across a range of frequencies which allows different parameters of the system to be examined. Small changes at the interface, such as the binding of a DNA target to a single stranded probe, are able to produce a large change in the impedance response, which gives this technique its high sensitivity. The ability to identify many characteristics of a system with high sensitivity was one of the key factors behind the use of EIS in this study.

The electrodes are also characterised using scanning electron microscopy (SEM) and atomic force microscopy (AFM) techniques to gain an impression of their individual surface profiles. We compare these characterisations to the final performance of each electrode when challenged with a clinically relevant antibiotic resistance gene sequence, amplified by polymerase chain reaction (PCR). We attempt to identify some characteristics of each electrode that play a key role in the overall sensitivity and specificity of the sensor. It is important to point out that, when searching the literature and planning these experiments, a great diversity of electrode types, surface preparation techniques, self-assembling monolayer (SAM) constituents and immobilisation conditions, and measurement approaches are reported alongside the development of electrochemical DNA biosensors. This study represents an attempt to identify some key factors which have a strong influence over the performance of such sensors.

## 2. Materials and Methods

Polycrystalline gold electrodes (PGEs) with a diameter of 2 mm were obtained from IJ Cambria (Llanelli, UK), and thin film gold electrodes (TFGE) of various sizes were obtained from FlexMedical Solutions (Livingston, UK). Gold SPEs with a 1.6 mm diameter working electrode were obtained from DropSens (Llanera, Spain) in two forms: AT (high-temperature cure) and BT (low-temperature cure). All solutions were prepared with deionised (DI) water (Scientific Laboratory Supplies, Nottingham, UK). PCR was performed using a HST+ DNA polymerase and associated reagents from Qiagen (Hilden, Germany). 6-Mercapto-1-hexanol (MCH), and 3-Mercapto-1-propanol (MCP) were obtained from Sigma Aldrich (Dorset, UK). All other chemicals were purchased from Acros Organics (Thermo Fisher Scientific, Geel, Belgium). The PCR product was generated by the amplification of antibiotic resistance gene sequences loaded onto an artificial plasmid. This plasmid was designed to harbour many Gram-negative antibiotic resistance genes for sensor development and testing, and to act as an accurate representation of how these genes would be encountered in a clinical setting. The AMR genes used in this study are the OXA-1 beta-lactamase (OXA) and a tetracycline efflux transporter (tetA). Table 1 provides the primer and probe sequences used in this work. PCR was performed according to the kit manufacturer’s instructions for 30 cycles and used without purification for DNA detection. The OXA amplicon was used as a complementary target, and the tetA amplicon was used as the non-complementary target.

Prior to functionalisation, all electrodes were cleaned thoroughly. PGEs were cleaned by immersion in Piranha solution, polished with alumina slurry, sonicated for 10 min in distilled H_2_O and then cycled by CV under 0.1M H_2_SO_4_ to fully regenerate the surface. This produces a smooth and uniform surface for SAM formation. SPEs and TFGEs cannot be cleaned using such harsh methods as the surfaces are easily damaged. These electrodes were therefore cleaned by CV cycling in H_2_SO_4_ until the gold oxide reduction peak no longer increased in size, which removes surface contaminants without damaging the gold surface. These differences in cleaning protocol are not expected to affect the results, as the impedance responses will be much more affected by the innate surface roughness and composition, which cannot be improved for SPEs and TFGEs.

Surface functionalisation was performed based upon work by Keighley et al. [26]. This protocol was chosen as it produced a consistent SAM layer on PGEs which was highly reproducible between experiments. Electrodes were incubated with 3 µM DNA probe and 30 µM alkanethiol in 0.8 M PBS + 1mM ethylenediaminetetraacetic acid (EDTA) overnight. Electrodes were then rinsed sequentially in 0.8 M PBS + 1 mM EDTA, 0.2 M phosphate buffer (PB), 10 mM PB and 10 mM PB + 10 mM EDTA (all pH 7). Electrodes were next backfilled with 1 mM alkanethiol in distilled water for 1 h and rinsed thoroughly with distilled water.

All measurements were performed using a three-electrode cell. The PGEs and TFGEs used a platinum counter electrode and a saturated Ag/AgCl reference electrode. When using SPEs, the counter electrode consisted of a cured gold ink of the same type as the working electrode, and the on-board silver electrode was used as reference. All measurements were performed using an Autolab PGSTAT204 potentiostat (Metrohm-Autolab, Utrecht, Netherlands) or a PalmSens PS4 potentiostat (PalmSens, Houten, Netherlands). Electrodes were equilibrated for 15 min under measurement solution before measurements were performed.

Clean electrodes were characterised by performing CV in measurement solution consisting of 50 mM PB + 200 mM KCl + 2 mM Fe[CN]_6_^3−^ + 2 mM Fe[CN]_6_^4−^ (pH 7). CV was performed between -0.2 V and +0.6 V at 0.1 V/s and was used to assess the nature of the response for each electrode. The Randles–Sevcik equation was used to calculate the real surface area of each electrode. Electrodes were also characterised in the same measurement solution using EIS. The EIS response was measured at frequencies between 100 kHz and 0.1 Hz, and the impedance spectra were fitted to a Randles equivalent circuit in order to determine the charge transfer resistance (Rct). Expected EIS responses to the stepwise modification of a macroelectrode surface are shown in Figure 1B, and inset is the Randles equivalent circuit used to fit the data. The Chi-squared goodness of fit and observation of the data determined whether the Randles equivalent circuit shown in Figure 1, or the simpler R(QR) circuit which excludes the Warburg impedance, would be used to fit the data.

Following electrode modification and backfilling, CV and EIS were performed in measurement solution to establish a baseline response for each sample. Electrodes were then rinsed thoroughly with 200 mM PB + 800 mM KCl (pH 7) before target DNA addition. Samples were incubated for 1 h at room temperature with 0.75µM DNA target in 200 mM PB + 800 mM KCl. Following this incubation, electrodes were rinsed in 200 mM PB + 800 mM KCl and 50 mM PB + 200 mM KCl, before final CV and EIS measurements were performed.

## 3. Results and Discussion

### 3.1. Electrode Performance—Initial Characterisation

**Figure 2:**
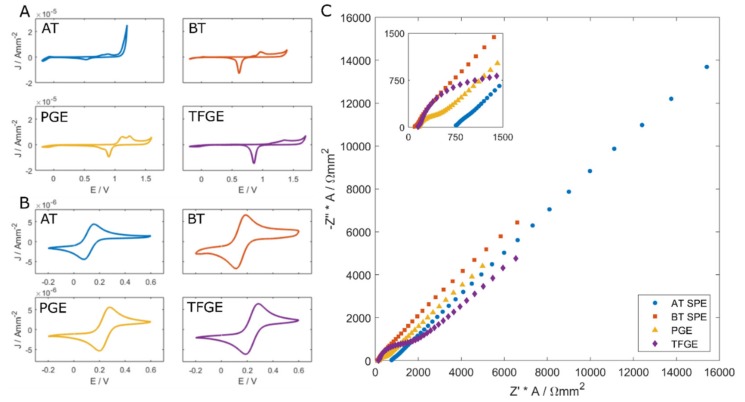
(**A**) Characterisation of clean polycrystalline gold electrode (PGE), thin-film gold electrode (TFGE) and clean AT and BT screen-printed electrodes (SPE). 0.1M H_2_SO_4_ CV cleaning responses. (**B**) CV and (**C**) impedance performed in 2 mM Fe[CN]_6_^3−^/Fe[CN]_6_^4−^ + 50 mM PB + 200 mM KCl. (**C**) Inset: Close up of high frequency impedance responses.

Characterisation of the clean electrodes was performed through electrochemical measurements and a physical examination of the surface. EIS and CV were employed to understand the initial electrochemical properties of each interface type. Figure 2 shows the cleaning CV as well as Fe[CN]_6_^3−^/Fe[CN]_6_^4−^ CV and impedance responses before any chemical modifications were made to the electrodes.

The cleaning CVs (Figure 2A) show typical responses for gold immersed in H_2_SO_4_. AT and BT SPEs show different gold oxide formation and reduction profiles which can be attributed to the different crystal faces predominating on the gold particles employed in the ink, possibly resulting from the different curing temperatures [27]. The CV voltage windows for each SPE type are also slightly different. AT electrodes produce a large oxygen evolution peak beyond +1.2 V, whereas BT electrodes produce this response above +1.4 V. The width of this potential window is related to the proportion of exposed gold, with higher proportions producing a narrower voltage window [28]. The cleaning CVs for the PGEs and TFGEs are similar, which is to be expected, as these both exhibit a pure gold surface. Small differences possibly arise from the effect of the different cleaning methods employed.

CVs in the presence of Fe[CN]_6_^3−^/Fe[CN]_6_^4−^ show a typical response for the redox couple (Figure 2B). The peak-to-peak separation of CV-cleaned AT SPEs was typically around 70 mV, whereas the BT SPEs showed approximately a 75 mV separation. The TFGEs exhibited peak-to-peak separations of around 90 mV, and the PGEs exhibited a separation of 73 mV. The SPEs and PGEs are close to the ideal peak separation of 59 mV at 298 K as described by the Nernst equation [25], indicating excellent reversibility of the system. Systems employing the TFGE are slightly less reversible after initial cleaning.

The CV data correlates well with the EIS responses of these electrodes under the same solution (Figure 2C). SPEs show rapid rates of electron transfer, indicated by the shape of the plot suggesting a high lambda value, and the fact that the Warburg impedance dominates even at high frequencies. The non-screen-printed gold surfaces show a small Rct feature, which shows that electron transfer is slowed at these surfaces. The TFGE exhibits a larger baseline Rct because the gold material is deposited thinly (approximately 10–20 nm) and therefore has a higher internal resistance than polycrystalline gold or screen-printed deposits.

**Figure 3:**
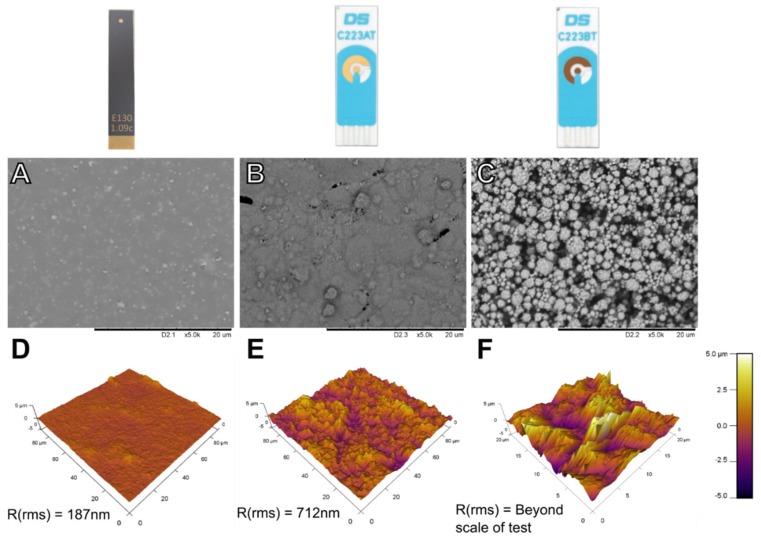
Microscopy images of different electrode types. Scanning electron microscopy images of (**A**) thin-film gold electrode, (**B**) AT and (**C**) BT screen-printed electrodes. Atomic force microscope surface topography scans of (**D**) thin-film gold electrode, (**E**) AT SPE and (**F**) BT SPE, with median R_rms_ from 5 different areas quoted.

Electrode surfaces were also visualized using microscopy and surface profiling to assess innate roughness. Scanning electron microscopy (SEM) images are shown in Figure 3A–C, and atomic force microscopy (AFM) images in Figure 3D–F. The TFGE has a highly smooth surface when viewed under both SEM and AFM. Of the five 90 µm × 90 µm areas examined on a fresh electrode, the median root mean squared roughness (R_rms_) was 187 nm.

The screen-printed sensors show a rougher profile, which is expected of a surface formed by the deposition of gold particles suspended in an ink. AT electrodes exhibit a reasonably smooth surface with isolated voids and raised regions on the micrometre scale. The median R_rms_ when examined under AFM was 712 nm, significantly higher than that of the TFGE. BT SPEs show an even more irregular surface than AT SPEs when examined under SEM, with deep voids of several micrometres in size throughout the surface and non-homogenous particle sizes. These electrodes could not be accurately imaged by AFM due to the large scale of the differences exceeding the capabilities of the measurement technique. It was not possible to examine the PGEs by SEM of AFM due to the size of the electrodes.

This initial characterisation of the electrodes suggests that SPE sensors would be the most suitable for DNA SAM formation in a point of care setting. Both SPE types showed very low initial Rct values and excellent reversibility of the Fe[CN]_6_^3−^/Fe[CN]_6_^4−^ redox couple. The TFGE had the largest Rct and the widest peak separation despite possessing the least rough surface of all the electrodes examined, which may indicate that it will not perform as well as the other electrode types as a DNA biosensor.

### 3.2. Electrode Performance Following Cleaning and Immobilisation of SAM Layers

Once cleaned and characterised, electrodes were incubated with a mixed SAM solution containing thiolated DNA probe and a short chain alkanethiol molecule (either MCH or MCP). Previous work by this group has successfully detected specific DNA binding using the techniques detailed here [29], and has optimised DNA target binding by examining the effect of DNA target length and overhang length [30], and the effect of agents such as tris(2-carboxyethyl)phosphine (TCEP) and formamide for electrode preparation and target incubation [31]. This work is therefore focussed on examining the role electrode surface conditions and SAM thickness have on biosensor performance to provide insight into how electrode and SAM selection can affect the sensitivity and specificity of DNA detection. 

**Figure 4:**
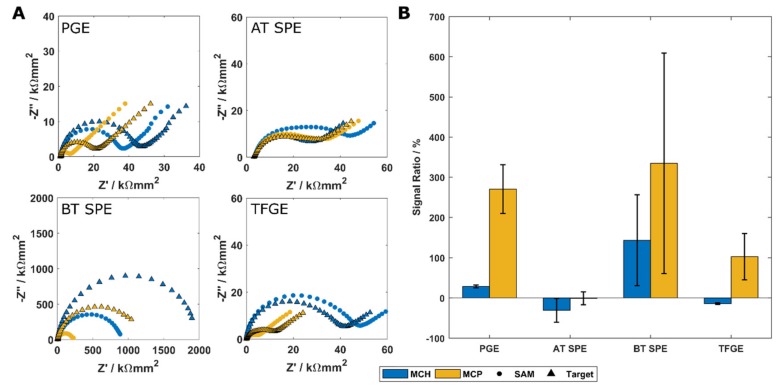
Impedance responses of polycrystalline gold electrodes, AT and BT SPEs, and thin-film gold electrodes. (**A**) Example area-corrected impedance responses of each electrode type with different SAM layers. (**B**) Mean percentage change in charge transfer resistance in response to DNA target binding on each electrode type. Error bars indicate standard deviation, n = 3.

EIS was performed to measure impedance changes upon the formation of a DNA SAM and complementary target binding. The signal ratio is calculated as the percentage change in Rct following DNA hybridisation (therefore, a 0% signal ratio indicates no change with DNA hybridisation relative to the pre-hybridisation Rct measured), and is the mean of all electrodes in a group. This is a common way of reporting Rct changes in response to analyte binding [26]. The signal increase after target hybridisation on each electrode type is presented in Figure 4.

Typical EIS responses for each electrode type are presented in Figure 4A. As expected, MCH SAMs produce a larger Rct than monolayers of MCP. This is due to the increased thickness of the MCH monolayer reducing the efficiency of electron tunnelling through the intact SAM, and is consistent across all electrode types [25,32]. In most cases, the addition of target DNA to an electrode caused an increase in the impedance of the electrode. This is the expected response on macroelectrodes when using a negatively charged redox couple such as Fe[CN]_6_^3−^/Fe[CN]_6_^4−^ due to increased steric hindrance and negative charge accumulation at the electrode surface [26,33]. The MCH SAM exhibited a smaller response increase than the MCP SAM, which is due to the lower initial Rct of the MCP SAM allowing a similar absolute change in Rct to produce a much larger signal ratio. This type of effect, i.e., improving sensitivity by reducing initial Rct, has also been reported through the use of PNA or morpholino-based probe molecules [29,34]. The use of a shorter alkanethiol allows a similar effect to be achieved at a lower cost but has its own limitations; shorter alkanethiols are less stable due to reduced intermolecular attractions binding the film together [35] and typically have more defects within the SAM [36]. DNA-sensing layers containing short alkanethiols may also show an ageing effect when the sensor is washed or regenerated [37,38]. Such an effect may be admissible in the laboratory, but for point-of-care testing and reusable sensors, this method of signal enhancement may not be appropriate.

The PGE and BT SPE both exhibit the expected behaviour in response to DNA target binding, with an increase in Rct observed independent of SAM composition. The BT SPE had significantly larger impedance responses than the PGE, which may be a result of the proportion of exposed gold being relatively low compared to other electrodes, resulting in a smaller conductive area [28]. Both the AT SPEs and the TFGEs showed similar impedance responses with an MCH SAM. The AT SPE shows an unexpectedly large impedance with an MCP SAM relative to the MCH SAM. There may be some effect from the surface roughness or ink composition here, causing the mixed monolayer to form in a way which produces a greater impedance than expected. This may be supported by the TFGE, which shows a similar MCH impedance response and a more typical MCP response.

Figure 4B shows the responses of each electrode and SAM combination to the addition of a complementary DNA target. The PGE produced consistent responses as expected. There are likely contributions to this consistency from the ability to clean these electrodes more thoroughly than the other types examined. The BT SPE showed a larger signal response than the PGE for both MCH and MCP SAMs, however in both cases this was highly variable. This is believed to be due to the complex surface topography allowing the DNA to take many orientations at the surface, rather than the highly organised and vertical SAM expected on more planar substrates. Although this surface could not be examined with AFM, it is expected that the surface will also be rough, which will increase the number of defects in the SAM layer and therefore increase variability. 

The AT SPE produced very little response from baseline with both MCH and MCP modification, and in both cases, a mean decrease in signal was observed following DNA target incubation. The TFGE exhibits a similar but smaller decrease in signal with an MCH SAM, and an increase in signal when a DNA target is added to an MCP SAM. As these surfaces are more similar to the PGE than the BT SPE surface, we might expect that these electrodes would give responses more like a PGE. The AT SPE appears relatively insensitive to the addition of DNA target with the protocol used, which may be a result of this method not being optimised for screen-printed surfaces. Both the AT SPE and TFGE showed a consistent decrease with an MCH SAM which may result from single-stranded DNA remaining close to the surface as it has a low persistence length, but opening channels through which the redox mediator may move when in the more rigid double stranded hybrid [33,39]. A positive signal ratio with MCP SAMs may result from reduced electrostatic attraction of the probe DNA to the electrode due to the DNA strand originating further out into solution from the SAM surface.

Based upon these results, for the simple transfer of protocols from traditional PGEs to a suitable PoC electrode, the TFGE appears to offer the best responses, as the responses of these electrodes are similar but smaller than the PGE. The BT SPE offers greater response sizes, but the variability in these electrodes was significant and is expected to remain high even with more electrodes tested due to electrode-to-electrode and batch-to-batch variation in the printing process. In contrast to this, the AT SPE offered acceptable reproducibility but showed minimal response to DNA target binding. The TFGE, especially with a shorter alkanethiol SAM, provided reasonable response sizes with enhanced reproducibility with only a small number of repeats. Whilst the results were not statistically significant, the experiments were self-consistent which allows comparison of the three electrode types (AT, BT and TFGE). In these comparisons, it was clear that the TFGE gave the response most similar to a PGE. In addition to this, the manufacturing process of the TFGE is much less variable and therefore batch-to-batch variability is expected to be low. While these SPEs may offer an excellent platform for biological measurement when using an optimised protocol [18,21,40], for rapid translation from the benchtop to bedside we believe TFGEs may offer the simpler solution.

### 3.3. Specificity and Sensitivity of a TFGE-Based Biosensor

**Figure 5:**
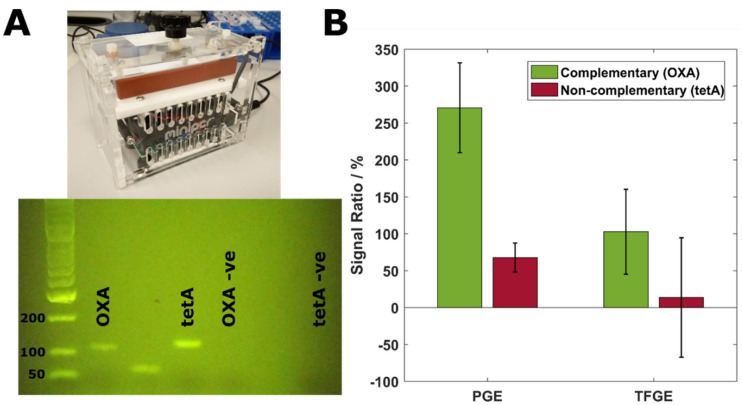
(**A**) Benchtop PCR thermocycler and gel electrophoresis results of complementary and non-complementary target amplification. –ve columns show amplification of a no-template control containing primers for the respective gene. (**B**) PGE and TFGE responses to the addition of complementary and non-complementary DNA target to electrode surface. Error bars indicate standard deviation, n = 3.

We next performed a specificity test on the TFGE biosensor to examine whether the performance of this system was similar to that of the PGE which we hope to replace. Electrodes were modified with an MCP SAM layer, as this showed the greatest signal-to-baseline ratio for both the PGE and TFGE, and then challenged with a non-complementary PCR amplicon of a similar length which was amplified from the same plasmid (the tetA sequence). Figure 5 shows the PCR outcome and the specificity results from this test.

A miniature thermocycler was used to amplify all PCR product used in these experiments. As shown in Figure 5A, this successfully amplified the two products of interest with no non-specific amplification or cross-contamination visible. When the PGE was challenged with complementary target DNA, a mean Rct increase of 270% was observed (Figure 5B). The addition of non-complementary target produced a mean increase of 68%, which is believed to be due to the target adsorbing at defects in the SAM layer rather than non-specific hybridisation. Much of this signal increase was lost when the protocol was transferred onto a TFGE, which showed only a 103% mean increase in Rct with complementary DNA. When non-complementary DNA was added, the response size was low at only 13%. However, in both cases, the variability of the measurement is high relative to the signal size. This is believed to be due to the limited cleaning processes available for the TFGE resulting in surfaces which are less consistent than is possible with the PGE. While these surfaces most closely resemble those of a PGE, and the responses reflect those achieved on PGEs, there may be further optimisation of cleaning or SAM formation required to reduce the variability of the signal, especially with regards to filtering non-specific signal increase. However, the mean response sizes are promising for the accurate discrimination of complementary vs. non-complementary DNA, and for filtering non-specific signals in more complex media such as a PCR reaction mix. We also believe that, based on data previously presented, the translation of this protocol onto SPEs would not produce such results without extensive optimisation. We suggest that using surfaces more similar to a PGE, while not producing an identical response, will speed up the development of DNA biosensor platforms when methods are initially developed on classic benchtop electrodes.

Whilst this study did not achieve statistical significance for the OXA vs. tetA experiment, it is clear that differential hybridisation was achieved. It is also important to note that the PCR reaction adds an additional level of specificity, only amplifying genes which are present in the sample and primed for. This means that in a real-world sensor, specificity would come largely from the PCR step. However, it is reassuring that the OXA probe has a good level of specificity at room temperature.

## 4. Conclusions

A number of different electrodes were examined by microscopy and electrochemical characterisation in order to assess their suitability as a platform for point-of-care DNA biosensing. The electrochemical characterisation of clean electrodes suggested that screen-printed electrodes would perform best, despite microscopic examination showing these to be much rougher than other electrodes tested—a characteristic which is known to have a detrimental effect on biosensor performance. This was exemplified by EIS investigation on AT and BT electrodes which showed minimal signal response or high variation, respectively, believed to be the result of inconsistent SAM formation on the rougher substrate. A thin-film gold electrode showed the greatest promise for use as a point-of-care sensor, with the smoother surfaces producing reasonable response sizes and reproducible results due to increased SAM uniformity. Electrodes fabricated with screen-printing technology are expected to require more optimisation of the cleaning and SAM formation protocols to obtain reproducible results than surfaces based on thin gold films, which appear to be sensitive to target binding using already well-established cleaning and SAM formation methods. Specificity testing suggested that thin-film gold electrodes provided similar results to classic polycrystalline gold with minimal optimisation. It is expected that thin-film gold electrodes would facilitate the easier transition of protocols from the benchtop to point-of-care testing as they better resemble the polycrystalline gold electrodes currently used during benchtop measurements. 

It was also noted that the use of the shorter alkanethiol molecule (MCP) produced a lower initial starting value for Rct and ultimately a more sensitive response compared to the longer variants, although at the cost of reproducibility. The use of a shorter SAM molecule increases the sensitivity of single-use devices, but would be inappropriate for reusable devices due to the instability of these monolayers over time. Altogether, these findings show that the careful control of surface topography and SAM composition are vital for the effective performance of electrochemical biosensors and should be given proper consideration when a sensor system is being developed. The ability, as shown here, to tune the surface properties on a relatively inexpensive sensor substrate points towards real-world applications of label-free SAM-based electrochemical biosensors.

## Figures and Tables

**Table 1:** DNA primer and probe sequences used in this work.

Sequence Name	Forward Sequence	Reverse Sequence	Amplicon Length (bp)
OXA Primers	AACAGAAGCATGGCTCGAAA	TGGTGTTTTCTATGGCTGAGTT	116
tetA Primers	GCATGATGAAGAAGACCGCCA	GAGTCGCACAAAGGCGAAC	121
DNA Probe	[ThiC6]AACAGAAGCATGGCTCGAAA		
